# A preoperative risk scoring system for survival prediction in clinical stage IB lung adenocarcinoma: a multicenter study

**DOI:** 10.3389/fonc.2025.1712276

**Published:** 2026-01-06

**Authors:** Kotaro Murakami, Tetsuya Isaka, Takuya Nagashima, Hiroyuki Adachi, Shunsuke Shigefuku, Noritake Kikunishi, Naoko Shigeta, Yujin Kudo, Yoshihiro Miyata, Morihito Okada, Norihiko Ikeda, Hiroyuki Ito

**Affiliations:** 1 Department of Thoracic Surgery, Kanagawa Cancer Center, Yokohama, Japan; 2 Department of Surgery, Tokyo Medical University, Tokyo, Japan; 3 Department of Surgical Oncology, Hiroshima University, Hiroshima, Japan

**Keywords:** lung adenocarcinoma, clinical stage IB, overall survival, scoring system, preoperative risk

## Abstract

**Background:**

Clinical stage (c-stage) IB lung adenocarcinoma (LUAD) presents variable survival outcomes, and the prognostic significance of factors such as ground-glass opacity components and positron emission tomography (PET) metrics remains unclear. Despite recent advances, no preoperative scoring model has been established to stratify risk in this subgroup. We aimed to identify preoperative prognostic factors in c-stage IB LUAD and develop a simple scoring system for predicting overall survival (OS).

**Methods:**

We retrospectively analyzed data from 245 patients with c-stage IB LUAD who underwent lobectomy at three institutions between 2010 and 2020. Cox regression analysis was performed to identify independent preoperative prognostic factors for OS. A risk score was developed by assigning points to each factor, based on the regression coefficients. Thereafter, patients were stratified into four risk groups based on their total scores.

**Results:**

Multivariate analysis identified smoking history (hazard ratio [HR]: 2.68; 95% confidence interval [CI]: 1.13–6.33; p=0.025), elevated serum carcinoembryonic antigen (CEA) levels (HR: 2.89; 95%CI: 1.42–5.91; p=0.004), and high maximum standardized uptake value (SUVmax) on PET (HR: 2.84; 95%CI: 1.16–6.98; p=0.023) as independent factors of poor prognosis. A scoring system was established by assigning one point to each factor. Patients were stratified into four risk groups: low (score 0, n=41), moderate (score 1, n=84), moderately high (score 2, n=77), and extremely high (score 3, n=43). Five-year OS rates were 100.0%, 89.3%, 74.0%, and 52.1%, respectively (p<0.001). The prognostic model demonstrated acceptable discrimination ability, with an area under the curve of 0.738 (95% CI, 0.661–0.815) and a concordance index of 0.753 (95% CI, 0.682–0.824). Notably, patients with a score of 0 showed low-grade tumors and favorable prognosis, whereas those with a score of 3 had more aggressive pathological characteristics and significantly worse outcomes.

**Conclusions:**

We developed and validated a simple preoperative scoring system using smoking history, serum CEA level, and tumor SUVmax to predict prognosis in c-stage IB LUAD. This model provides a practical tool for risk stratification and may support individualized treatment decisions, including the consideration of induction therapy in selected cases.

## 1 Introduction

Lung cancer is a leading cause of morbidity and mortality worldwide. Among lung cancer cases, lung adenocarcinoma (LUAD) accounts for over 60% of cases, making it the most common histological subtype, particularly in Japan (1).

The Union for International Cancer Control (UICC) tumor-node-metastasis (TNM) staging system is the only established tool for stratifying recurrence risk in lung cancer (2). Recently, ground-glass opacity (GGO) components and the maximum standardized uptake value (SUVmax) on positron emission tomography (PET) have emerged as important prognostic factors in clinical stage (c-stage) I LUAD (3–5).

Following the TNM classification update to the 8th edition, several studies have explored potential preoperative prognostic factors in c-stage IA non-small cell lung cancer (NSCLC). Hattori, et al. (6) reported that the presence of a GGO component in stage IA NSCLC is associated with favorable prognosis. Similarly, a large meta-analysis of patients with c-stage IA NSCLC showed that part-solid tumors had a better prognosis than pure-solid tumors (7). Additional studies have identified PET imaging as a significant prognostic tool in this population (8, 9). Although numerous studies have investigated prognostic factors for c-stage IA NSCLC, the prognostic relevance of GGO components and PET metrics in c-stage IB NSCLC remains unclear. To the best of our knowledge, no study has specifically analyzed the prognostic significance of preoperative factors, such as GGO components or SUVmax, in c-stage IB NSCLC (TNM 8th edition) or developed a risk stratification model for this subgroup.

Lobectomy with lymph node dissection is the standard treatment for c-stage IB lung cancer (10). However, the 5-year overall survival (OS) rate for patients with c-stage IB (T2aN0M0) NSCLC remains unsatisfactory, ranging from 68 to 71.5% (11, 12). In recent years, several trials have evaluated the efficacy of neoadjuvant chemotherapy and immunotherapy, showing therapeutic benefits (13, 14). However, these trials included only a limited number of patients with c-stage IB (TNM 8th edition), highlighting the need for further studies to identify eligible candidates. Notably, a subset of patients with c-stage IB LUAD could potentially benefit from preoperative treatment. Identifying and scoring prognostic factors could enable better risk stratification, guiding clinical decision-making, such as optimizing treatment selection and identifying candidates for induction therapy. Preoperative risk stratification may help tailor individualized treatment strategies to improve patient outcomes.

Importantly, the relevance of GGO components and SUVmax, which have been established as preoperative prognostic factors for c-stage IA LUAD, in c-stage IB LUAD remains unclear. Moreover, GGO components are specific to LUAD, whereas non-adenocarcinomas typically present as pure-solid tumors. Additionally, SUVmax has been reported to be significantly higher in non-LUADs than in LUADs (15). Therefore, the present study focused exclusively on patients with c-stage IB LUAD. Specifically, this study aimed to identify preoperative prognostic factors for c-stage IB LUAD and develop a simple scoring tool for preoperative risk prediction.

## 2 Methods

### 2.1 Patients

A retrospective chart review was performed using our prospectively maintained database to identify patients who underwent surgical resection for primary LUAD at Kanagawa Cancer Center, Tokyo Medical University, and Hiroshima University Hospital between January 2010 and December 2020. The study was approved by the Institutional Review Board of Kanagawa Cancer Center (24 Eki, 54), with a waiver for written informed consent.

Medical record data were updated in May 2023 and extracted based on clinicopathological features and treatment histories. Patients without documented clinical or radiographic disease progression were censored at their last follow-up visit. Patients with non-adenocarcinoma or those who underwent sublobar resection (wedge resection or segmentectomy) were excluded. All included patients underwent preoperative high-resolution computed tomography (CT) and fluorodeoxyglucose-PET/CT (FDG-PET/CT). Disease staging was determined according to the 8th edition of the UICC TNM classification for lung and pleural tumors (2). Complete resection was defined as the absence of residual cancer, either macroscopically or microscopically.

### 2.2 Patient follow-up

Follow-up evaluations included physical examination, chest radiography, chest CT, and blood tests for relevant tumor markers. Follow-up chest CT was performed every 6–12 months. Additional diagnostic evaluations were conducted, including CT scans of the chest and abdomen, brain magnetic resonance imaging (MRI), and FDG-PET/CT, if recurrence was suspected.

### 2.3 FDG-PET/CT assessment

FDG-PET/CT scans were performed using one of the following integrated three-dimensional PET/CT scanners: Discovery MI (GE Healthcare, Little Chalfont, United Kingdom), Aquiduo (Toshiba Medical Systems Corporation, Tochigi, Japan), and Biograph Sensation 16 (Siemens Healthcare, Erlangen, Germany). Additionally, the SUVmax values were standardized across the three institutions using the method proposed by Nakayama et al. (16). Radiologists at each institution independently determined the original SUVmax.

### 2.4 Pathological assessment

All collected surgical specimens were fixed in 10% formalin and embedded in paraffin blocks. DNA was analyzed for epidermal growth factor receptor (EGFR) mutations using the Cobas^®^ EGFR Mutation Test version 2 (Cobas; Roche Diagnostics, Basel, Switzerland) (17). Tumor differentiation was classified as well-differentiated (minimally invasive adenocarcinoma or lepidic), moderately differentiated (acinar or papillary), or poorly differentiated (solid or micropapillary) adenocarcinoma (18).

### 2.5 Statistical analysis

Relapse-free survival (RFS) and OS were estimated using the Kaplan–Meier method, and differences in survival rates were assessed using log-rank tests. RFS was defined as the time from surgery to recurrence or death from any cause. OS was defined as the time from surgery to death or the last follow-up for censored patients (those without adverse events during the last observation period). For cutoff values for the variables, age was set at 75 years, the threshold for the elderly population, and serum carcinoembryonic antigen (CEA) was set at 5 ng/mL, upper limit of the normal range. Cutoff values for SUVmax were determined using receiver operating characteristic (ROC) curve analysis with RFS as the outcome based on the Youden index. Survival curves were constructed using the Kaplan–Meier method.

Univariate and multivariate analyses were performed using a Cox proportional hazards model, incorporating age, sex, smoking history, serum CEA level, CT tumor consolidation size, presence of pure solid tumors on imaging, and SUVmax value. Smoking history was included because of its reported association with cancer grade (19). To assess potential correlations between continuous variables, such as CEA, SUVmax, and CT tumor consolidation size, correlation coefficients were calculated, and no significant correlations were observed (all |r| < 0.3). Multivariate analysis was performed using the backward elimination method. Statistical significance was set at *p* < 0.05. All statistical analyses were performed using EZR on R Commander version 1.30 (Saitama Medical Center, Jichi Medical University, Saitama, Japan), a graphical user interface for R (R Foundation for Statistical Computing, Vienna, Austria).

### 2.6 Development of the scoring system

A risk-scoring tool was developed to predict the prognosis of patients with c-stage IB LUAD. Each independent preoperative predictor was assigned a point value based on the regression coefficients derived from the multivariate analysis. Patients were categorized into four risk groups: low (score 0), moderate (score 1), moderately high (score 2), and extremely high (score 3). The accuracy of the scoring system was evaluated using ROC analysis and the Cox proportional hazards model (20). Additionally, to validate the prognostic performance of the Cox proportional hazards model, we performed a three-fold cross-validation. Model discrimination was evaluated using the concordance index (C-index), calculated for each fold and averaged across all folds.

## 3 Results

**Figure 1:**
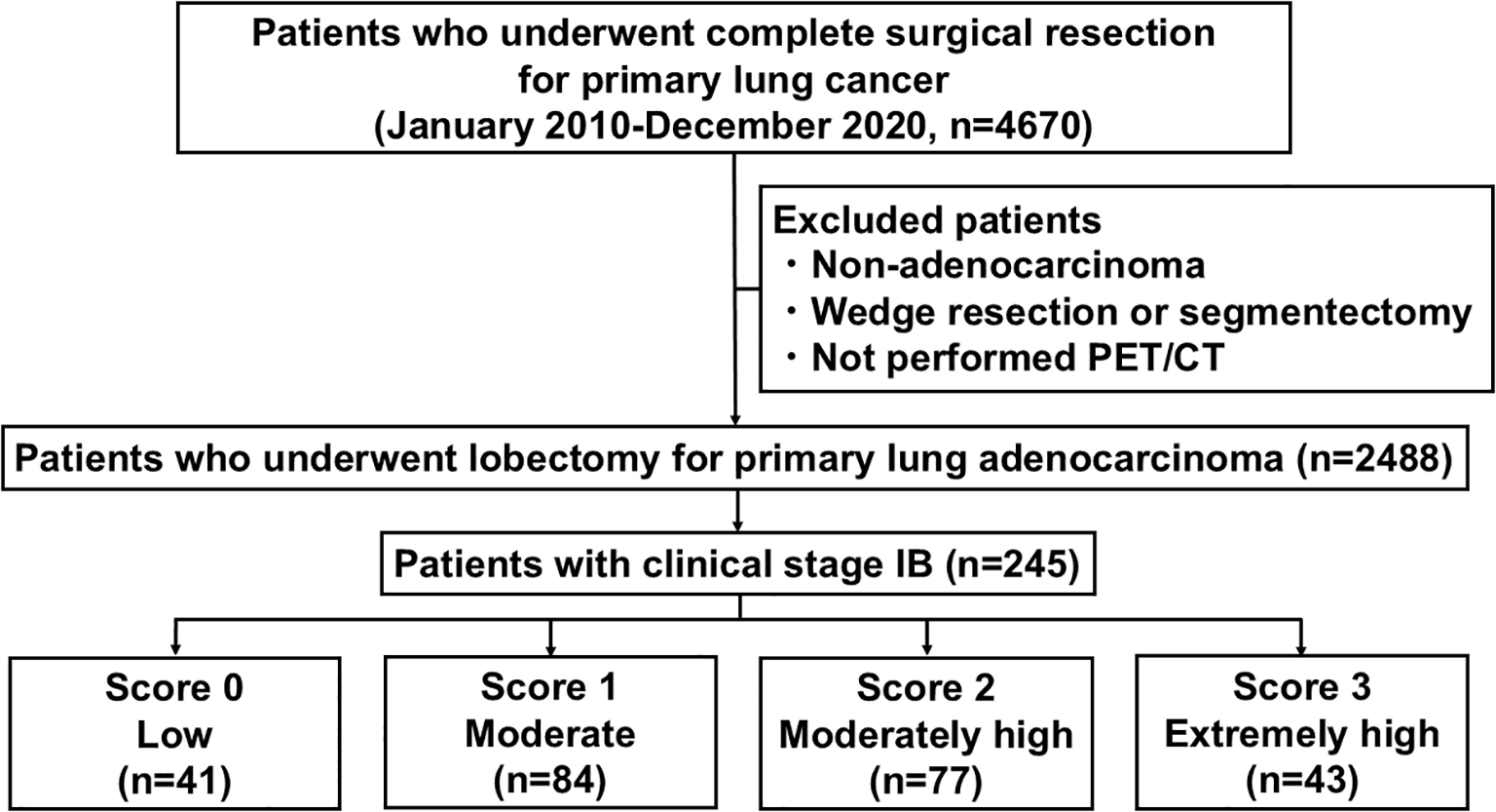
Flow diagram of the study design.

In total, 4,670 consecutive patients with primary lung cancer underwent complete surgical resection. Among the 2,488 patients who underwent lobectomy, 245 with c-stage IB disease were included in this study (**Figure 1**). Additionally, the median follow-up time for survivors was 45.0 months (range, 0.5–133.3). **Table 1** summarizes the patient characteristics. Among all patients, 55.1% had a history of smoking and 31.8% had elevated preoperative serum CEA levels. Additionally, the median tumor SUVmax was 4.7 (range: 0.0–34.7), with 36.3% of patients having pathological stage (p-stage) II–III disease. Multivariate analysis identified elevated CEA levels (hazard ratio [HR], 2.26; 95% confidence interval [CI], 1.46–3.51; *p* < 0.001) and SUVmax (HR, 4.60; 95% CI, 2.48–8.55; *p* < 0.001) as independent predictors of RFS (**Table 2**). Moreover, smoking history (HR, 2.68; 95% CI, 1.13–6.33; *p* = 0.025), elevated CEA levels (HR, 2.89; 95% CI, 1.42–5.91; *p* = 0.004), and SUVmax (HR, 2.84; 95% CI, 1.16–6.98; *p* = 0.023) were identified as independent predictors of OS (**Table 3**). Notably, the cutoff value for SUVmax was determined to be 3.7 (area under the curve [AUC], 0.713; 95% CI, 0.648–0.779) based on ROC curve analysis, with RFS as the outcome variable.

**Table 1:** Patient characteristics (n = 245).

Variables	Total (n = 245)
Preoperative findings	
Median age, years (IQR)	71.0 (65–77)
Sex, male (%)	139 (56.7)
Smoking history, yes (%)	135 (55.1)
Serum CEA level, (%)	
≦5 ng/mL	167 (68.2)
>5 ng/mL	78 (31.8)
Median CT tumor size, cm (IQR)	3.6 (3.3–3.9)
Median CT tumor consolidation size, cm (IQR)	3.4 (3.2–3.7)
Radiological tumor component, (%)	
pure solid tumor	175 (71.4)
part solid tumor	70 (28.6)
Median SUVmax (IQR)	4.7 (2.9–9.3)
Postoperative and pathological findings	
Tumor differentiation, (%)	
well	38 (15.5)
moderate	165 (67.3)
poor	42 (17.2)
Median pathological invasive tumor size, cm (IQR)	3.1 (2.1–3.7)
Lymphatic permeation, positive (%)	80 (32.7)
Vascular invasion, positive (%)	111 (45.3)
Visceral pleural invasion, positive (%)	89 (36.3)
Nodal metastasis, (%)	
pN0	191 (78.0)
pN1	32 (13.0)
pN2	22 (9.0)
Pathological stage in the eighth edition, (%)	
IA	75 (30.6)
IB	81 (33.1)
II-III	89 (36.3)
EGFR mutation, (%)	
positive	118 (55.9)
wild type	93 (44.1)
NA	34
Recurrence, yes (%)	70 (28.6)
Cause of death	
lung cancer recurrence	20
other causes	17

IQR, interquartile range; CEA, carcinoembryonic antigen; CT, computed tomography; EGFR, epidermal growth factor receptor; SUVmax, maximum standardized uptake value.

**Table 2:** Univariable and multivariable analyses for relapse-free survival in all patients with clinical stage IB adenocarcinoma.

	Univariable analysis			Multivariable analysis		
	Raw coefficients	Hazard ratio (95% CI)	*p*-value	Adjusted coefficients	Hazard ratio (95% CI)	*p*-value
Age, ≥75 years	0.34	1.41 (0.91–2.18)	0.124			
Sex, male	0.22	1.25 (0.80–1.95)	0.327			
Smoking history, yes	0.22	1.25 (0.80–1.94)	0.322			
Serum CEA level, >5 ng/mL	0.98	2.67 (1.73–4.13)	<0.001	0.82	2.26 (1.46–3.51)	<0.001
CT tumor consolidation size	0.17	1.19 (0.57–2.49)	0.643			
Radiological tumor component, pure solid tumor	0.45	1.57 (0.93–2.65)	0.093			
SUVmax, ≥3.7	1.63	5.10 (2.76–9.43)	<0.001	1.53	4.60 (2.48–8.55)	<0.001

CEA, carcinoembryonic antigen; CT, computed tomography; SUVmax, maximum standardized uptake value; CI, confidence interval.

**Table 3:** Univariable and multivariable analyses for overall survival in all patients with clinical stage IB adenocarcinoma.

	Univariable analysis			Multivariable analysis		
	Raw coefficients	Hazard ratio (95% CI)	*p*-value	Adjusted coefficients	Hazard ratio (95% CI)	*p*-value
Age, ≥75 years	0.61	1.84 (0.94–3.61)	0.076			
Sex, male	0.94	2.56 (1.15–5.66)	0.021			
Smoking history, yes	1.24	3.46 (1.50–7.99)	0.004	0.98	2.68 (1.13–6.33)	0.025
Serum CEA level, >5 ng/mL	1.37	3.92 (1.97–7.80)	<0.001	1.06	2.89 (1.42–5.91)	0.004
CT tumor consolidation size	1.00	2.72 (0.88–8.43)	0.083			
Radiological tumor component, pure solid tumor	0.14	1.15 (0.54–2.46)	0.722			
SUVmax, ≥3.7	1.27	3.57 (1.48–8.62)	0.005	1.04	2.84 (1.16–6.98)	0.023

CEA, carcinoembryonic antigen; CT, computed tomography; SUVmax, maximum standardized uptake value; CI, confidence interval.

**Figure 2:**
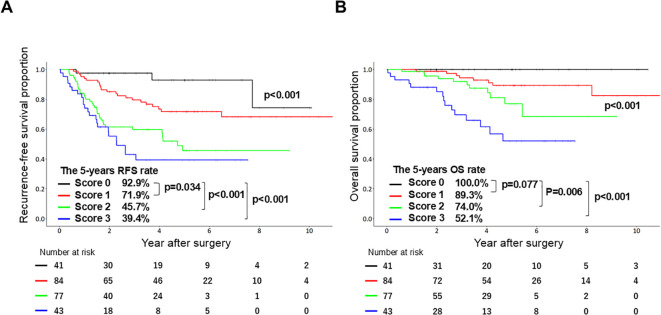
Survival curves. **(A)** Kaplan–Meier curves of relapse-free survival (RFS) in patients with clinical stage IB lung adenocarcinoma stratified according to the prognostic scoring system. **(B)** Kaplan–Meier curves of overall survival (OS) in patients with clinical stage IB lung adenocarcinoma based on the prognostic scoring system.

A risk-scoring system was developed based on the independent preoperative predictors of OS identified in the multivariate analysis. Specifically, the regression coefficients (adjusted coefficients; **Table 3**) were rounded to the nearest integer to assign point values to each predictor. Importantly, the scoring system stratified patients into four risk groups: scores 0 (n = 41), 1 (n = 84), 2 (n = 77), and 3 (n = 43). Kaplan–Meier survival curves demonstrated a significant decline in both RFS (*p* < 0.001; **Figure 2A**) and OS (*p* < 0.001; **Figure 2B**) with increasing risk scores. Notably, the 5-year OS rates for risk scores of 0, 1, 2, and 3 were 100.0, 89.3, 74.0, and 52.1%, respectively. **Table 4** shows the characteristics of the patients according to the risk score. Patients with a risk score of 0 had significantly better tumor differentiation (36.6%, *p* < 0.001), smaller median pathological invasive tumor size (2.1 cm, *p* < 0.001), and lower rates of vascular and visceral pleural invasion (VPI; 14.6 and 4.9%, respectively; *p* < 0.001). Only one patient (2.4%) in this group had pathological lymph node metastasis (*p* = 0.037). In this group, the median SUVmax was 2.0, and 61.0% of the patients had p-stage IA LUAD. In contrast, patients with a risk score of 3 exhibited larger pathological invasive tumor size (median value: 3.5, *p* < 0.001), higher SUVmax (median value: 10.1, *p* < 0.001), and more frequent vascular invasion and VPI (65.1 and 51.2%, respectively; both *p* < 0.001), with 53.5% of patients having p-stage II–III disease.

**Table 4:** Patient characteristics stratified by the prognostic scoring system.

Variables (n = 245)	Score 0 Low (n = 41)	Score 1 Moderate (n = 84)	Score 2 Moderately high (n = 77)	Score 3 Extremely high (n = 43)	*p*-value
Preoperative findings					
Median age, years (IQR)	71.0 (64.3–76.8)	71.5 (67–76)	72.0 (64–77)	70.0 (65–77)	0.993
Sex, male (%)	32 (78.0)	44 (52.4)	22 (28.6)	35 (81.4)	<0.001
Smoking history, yes (%)	0 (0.0)	36 (42.9)	56 (72.7)	43 (100.0)	<0.001
Serum CEA level, ≥5 ng/mL (%)	0 (0.0)	8 (9.5)	30 (39.0)	43 (100.0)	<0.001
Median CT tumor size, cm (IQR)	3.7 (3.5–4.1)	3.5 (3.2–3.9)	3.4 (3.2–3.8)	3.7 (3.4–3.9)	0.029
Median CT tumor consolidation size, cm (IQR)	3.44 (3.2–3.7)	3.3 (3.2–3.5)	3.4 (3.2–3.7)	3.7 (3.4–3.9)	<0.001
Radiological tumor component, (%)					<0.001
pure solid tumor	22 (53.7)	52 (61.9)	62 (80.5)	39 (90.7)	
part solid tumor	19 (46.3)	32 (38.1)	15 (19.5)	4 (9.3)	
Median SUVmax (IQR)	2.0 (1.5–3.0)	3.5 (2.5–6.5)	7.5 (4.5–9.8)	10.1 (6.0–14.7)	<0.001
Postoperative and pathological findings					
Tumor differentiation, (%)					<0.001
well	15 (36.6)	13 (15.5)	7 (9.1)	3 (7.0)	
moderate	26 (63.4)	65 (77.4)	45 (58.4)	29 (67.4)	
poor	0 (0)	6 (7.1)	25 (32.5)	11 (25.6)	
Median pathological invasive tumor size, cm (IQR)	2.1 (1.3–3.3)	2.9 (2.0–3.5)	3.2 (2.6–3.8)	3.5 (3.0–3.9)	<0.001
Lymphatic permeation, positive (%)	7 (17.1)	30 (35.7)	27 (35.1)	16 (37.2)	0.124
Vascular invasion, positive (%)	6 (14.6)	30 (35.7)	47 (61.0)	28 (65.1)	<0.001
Visceral pleural invasion, positive (%)	2 (4.9)	26 (31.0)	33 (42.9)	22 (51.2)	<0.001
Nodal metastasis, (%)					0.037
pN0	40 (97.6)	66 (78.6)	54 (70.1)	31 (72.1)	
pN1	0 (0.0)	10 (11.9)	14 (18.2)	8 (18.6)	
pN2	1 (2.4)	8 (9.5)	9 (11.7)	4 (9.3)	
Pathological stage in the 8^th^ edition, (%)					<0.001
IA	25 (61.0)	29 (34.5)	16 (20.8)	5 (11.6)	
IB	11 (26.8)	28 (33.3)	27 (35.1)	15 (34.9)	
II-III	5 (12.2)	27 (32.1)	34 (44.2)	23 (53.5)	
Recurrence, yes (%)	3 (7.3)	20 (23.8)	30 (39.0)	17 (39.5)	<0.001
Cause of death					
lung cancer	0	6	5	8	
other causes	0	2	6	7	

IQR, interquartile range; CEA, carcinoembryonic antigen; CT, computed tomography; SUVmax, maximum standardized uptake value.

The proposed preoperative prognostic scoring system demonstrated favorable predictive performance based on the ROC curve (AUC, 0.738; 95% CI, 0.661–0.815) and Cox proportional hazards model (C-index, 0.753; HR, 2.85; 95% CI, 1.923–4.226; *p* < 0.001). Furthermore, the results of the three-fold cross-validation showed a C-index of 0.727 ± 0.06 for RFS and 0.759 ± 0.039 for OS, indicating that the model’s discriminatory ability was relatively good.

## 4 Discussion

This study aimed to identify preoperative prognostic factors for c-stage IB LUAD and develop a simple and effective scoring system for preoperative prognostic prediction. In addition to SUVmax, smoking history and elevated CEA levels were identified as significant preoperative prognostic factors. Although the presence of GGO components is a well-established favorable prognostic factor in c-stage IA, it was not associated with prognosis in this cohort of patients with c-stage IB (3, 6). Notably, we developed a scoring system that effectively stratified postoperative outcomes by incorporating smoking history, elevated CEA levels, and SUVmax values. Additionally, the validity of this scoring system was confirmed, highlighting the heterogeneous nature of c-stage IB LUAD and the challenges in accurately predicting patient outcomes. To the best of our knowledge, this is the first study to evaluate preoperative prognostic factors and propose a scoring system specifically designed for c-stage IB LUAD (TNM 8th edition) following complete resection.

The relationship between tobacco smoking and lung cancer development is well established, with recent studies showing that smokers have a significantly poorer prognosis than non-smokers, particularly in early-stage LUAD (21). Sakao, et al. (19) reported that cigarette smoking is associated with the carcinogenesis of moderately to poorly differentiated LUAD, including papillary, acinar, and solid component subtypes. CEA is widely recognized as a valuable biomarker for diagnosing and monitoring lung cancer prognosis (22). A retrospective study found that elevated CEA levels were associated with poorer survival outcomes and served as a risk factor for occult regional lymph node metastasis in patients with stage I NSCLC undergoing surgical resection (23). Moreover, the preoperative SUVmax in the primary tumor of patients with c-stage I disease is associated with disease-free survival and OS (4, 5). In this study, preoperative smoking history, elevated CEA levels, and high SUVmax were identified as significant prognostic factors. Therefore, incorporating these factors into a scoring system provides a clinically practical tool for predicting the prognosis of c-stage IB LUAD. Patients classified into higher-risk groups by our scoring system exhibited larger pathological invasive tumor sizes and higher rates of poor tumor differentiation, vascular invasion, VPI, and lymph node metastasis.

In this study, GGO components were not significant prognostic factors. Aokage et al. (3) reported that approximately 20% of c-stage IB LUAD cases with a GGO component were classified as invasive solid-predominant adenocarcinoma and that the presence of the GGO component was not attributable to the prognosis of this cancer subtype. This may be because the increasing tumor diameter and associated malignant progression outweigh the potential prognostic benefits of the GGO component at this stage.

Previously reported 5-year OS rates for patients with c-stage IB (T2aN0M0) NSCLC ranged from 68 to 71.5% (11, 12). Although previous studies did not stratify prognosis based on preoperative factors, our study demonstrated that preoperative factors, such as smoking history, CEA levels, and SUVmax values, effectively stratified postoperative outcomes in c-stage IB LUAD. Notably, patients with a risk score of 0 had a 5-year OS rate of 100%, comparable to that of c-stage IA1 reported in previous studies (11, 12). Importantly, none of these patients (with a score of 0 on the present scoring system) showed pathologically poor tumor differentiation, and they exhibited lower frequencies of lymphatic permeation, vascular invasion, and VPI (17.1, 14.6, and 4.9%, respectively). Collectively, these results suggest that this scoring system is effective for discriminating low-grade tumors in patients with c-stage IB LUAD. Furthermore, only one patient (2.4%) in this group had lymph node metastasis, supporting the possibility of reduced lymphadenectomy. In contrast, patients with a risk score of 3 had significantly worse outcomes, with a 5-year OS rate of 52.1%, equivalent to that of c-stage IIB–IIIA reported in previous studies (11, 12). This high-risk group was characterized by a greater pathological invasive tumor size, more frequent vascular invasion, VPI, and a higher proportion (53.5%) of p-stage II–III disease. Considering their poor prognosis, these patients may benefit from neoadjuvant therapy. Future prospective multicenter validation of this preoperative risk scoring system is needed to explore strategies to improve survival outcomes in patients with extremely high-risk c-stage IB LUAD under the current TNM classification (8th edition).

Conclusively, the scoring system developed in this study effectively stratified the pathological grade and prognosis of c-stage IB LUAD based on preoperative factors, highlighting its heterogeneous nature. Incorporating this stratification into ongoing discussions regarding perioperative treatment, including preoperative induction therapy, may help refine clinical decision-making. Given its simplicity and reliance on preoperatively available parameters, this model can be readily incorporated into routine preoperative assessments and multidisciplinary discussions for stage IB LUAD.

Despite these promising findings, this study had some limitations, such as its retrospective nature, which may have introduced potential bias. Additionally, the statistical analyses may not be sufficiently robust to identify the effects of certain factors, such as sex. Moreover, the long duration of the study may contribute to substantial sample heterogeneity. Although the cutoff values for SUVmax were calculated using ROC curves and efforts were made to harmonize SUVmax values by correcting inter-facility errors, discrepancies in SUVmax measurements between institutions remain a concern. Moreover, no standardized surveillance protocol was established across the three participating institutions, either postoperatively or at recurrence, which may have affected the consistency of data collection and outcomes. Owing to the small sample size, this scoring system could not be validated using test data. Further prospective studies with larger multi-institutional cohorts are necessary to validate our findings. Due to the multicenter nature of this study, detailed quantification of smoking exposure, such as pack-years, could not be uniformly assessed in all the patients. As a result, smoking intensity was not rigorously evaluated, and only a binary classification (smoker vs. non-smoker) was adopted. Given that EGFR mutation testing was not performed in all patients, EGFR status was excluded from the multivariate analysis, even though it may influence treatment decisions, particularly for preoperative therapy. Moreover, the EGFR mutation status was determined via preoperative biopsy in some cases and surgical specimens in others, which could introduce variability. Future analyses incorporating molecular data are necessary. Although GGO was included as a categorical variable (part-solid vs. pure-solid), the consolidation-to-tumor ratio, a quantitative index used in other studies, was excluded due to potential inter-institutional variability in measurement. Despite these limitations, this study successfully identified preoperative prognostic factors for c-stage IB LUAD, marking an important step toward improving preoperative evaluation and treatment strategies.

## 5 Conclusions

Our findings suggest that smoking history, serum CEA level, and tumor SUVmax are critical determinants of prognosis in patients with c-stage IB LUAD, regardless of solid component size or the presence of a GGO component. The scoring system developed using these preoperative factors, in combination with the TNM classification of lung cancer, may enhance prognostic accuracy and aid in clinical decision-making for patients with c-stage IB LUAD.

## Data Availability

The raw data supporting the conclusions of this article will be made available by the authors, without undue reservation.
